# Proposal of Design and Innovation in the Creation of the Internet of Medical Things Based on the CDIO Model through the Methodology of Problem-Based Learning

**DOI:** 10.3390/s22228979

**Published:** 2022-11-20

**Authors:** Jefferson Sarmiento-Rojas, Pedro Antonio Aya-Parra, Oscar J. Perdomo

**Affiliations:** Biomedical Engineering Program, School of Medicine and Health Sciences, Universidad del Rosario, Bogota 111711, Colombia

**Keywords:** CDIO educational model, Internet of Things, problem-based learning, real-time monitoring

## Abstract

The educational framework—Conceive, Design, Implement, and Operate—is part of an international proposal to improve education in the field of engineering, emphasizing how to teach engineering comprehensively, which allows the standardization of skills in professionals as a model for teaching engineering. Moreover, problem-based learning allows students to experiment with challenging situations through cases that simulate natural contexts with their profession. The integration of these two education strategies applied to the Internet of Things (IoT) Education for Industry 4.0 has promoted the generation of teaching challenges. Our education strategy proposes the synergy between laboratory guides and the classroom with the following actions: the content of the topic is presented, followed by the presentation of an issue focused into a realistic context, with practical exercises integrating software and hardware for the deployment of the solution to be reported as a final project. Moreover, undergraduate students in the biomedical engineering area acquired new knowledge about IoT, but at the same time, they may develop skills in the field of programming and structuring different architectures to solve real-world problems. Finally, traditional models of education require new teaching initiatives in the field of biomedical engineering concerning the current challenges and needs of the labor market.

## 1. Introduction

The Internet of Things or simply termed IoT has become a global intercommunication network between people and objects in everyday life. It supports communications between electronic devices such as desktop computers, laptops, tablets, and mobile phones. Currently, millions of people in the world have at least one smart device capable of accessing the Internet, which has implications for the activities of daily life, work, and business [1]. Since the beginning of the technological revolution, the Internet has played a leading role in the development of industries, based on the interconnection of things. In this interconnection of objects and people, technologies are created to improve the quality of life of people; some examples of these are fitness bands, pulse monitors, clothing, and smart accessories, and all these devices are known as wearable smart devices.

The health sector takes advantage of the IoT to improve access to health, quality of care, consumer experience, and operational efficiency in hospitals and care centers. The Internet of Healthcare Things (IoHT) acquires physical signals from objects located in contexts related to the health area and transforms them into simple data that, through algorithms, can be transformed into relevant information for the health sector, which allows communication with other objects, medical devices, patients, and healthcare personnel. These interconnected devices are transforming the provision of medical services, introducing new monitoring and data collection systems, and promoting an improvement in the different processes related to the area of health. As a result of the interaction between devices, the IoT may support decision-making. According to estimates, the value of IoHT exceeded USD 163 billion by 2020, with a compound annual growth rate of 38.1% between 2015 and 2022. By next year, the healthcare sector is projected to be number 1 in the top 10 industries for the development of IoHT systems [2].

Healthcare providers and users invest in IoHT programs in three business areas: remote patient monitoring (RPM), wellness/prevention, and operations. The value of IoHT programs is evidenced in medical, administrative/operational cost savings, improvements in the consumer experience, and revenue growth through user attraction/retention [3].

With the evolution of technology and the new fields of knowledge that arise, the current engineering programs are dynamically transformed by the evolution of technology and innovation in recent decades. This has generated economic growth that has allowed the emergence of new paradigms in academia such as the “knowledge society” to describe socioeconomic evolution [4].

At present, the different teaching–learning activities planned and developed in engineering are the basis for generating new capacities for scientific thinking that allow the production and implementation of technological Research, Development, innovation (R+D+i) projects crucial in the training of new engineers [5]. 

In this context, the future engineer should be able to design mathematical models, understand the behavior of systems, have experimental skills, design processes, and integrate knowledge to solve real-life problems. To achieve this professional profile, it is relevant to develop and implement pedagogical strategies that allow the generation of new knowledge and skills in engineers. Generating changes in the training of engineering students is an essential need for the continual improvement of competitiveness, given the systematic changes that society faces in the health sector.

**Figure 1:**
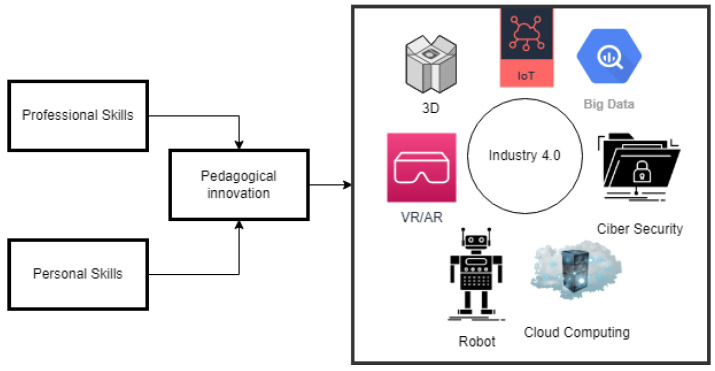
Personal and professional skills through educational pedagogy.

Under this scenario, the biomedical engineering program in the School of Medicine and Health Sciences (EMCS) of the Universidad del Rosario, in agreement with the Colombian School of Engineering Julio Garavito, participated in and was awarded, in the year 2019, the pedagogical innovation funding program “Nora Pabón Tovar” from the Universidad del Rosario, with the proposal titled “Teaching the future in the present: Internet of Things and its applications in health: A pilot study”, as it is presented in Figure 1.

The proposal directly relates personal and professional skills through an interesting educational pedagogy that was initially raised and designed under two fundamental paradigms, which were the CDIO initiative that provides an innovative framework for engineering education and focuses on the fundamentals of engineering framed in the context of conceiving, designing, implementing, and operating systems, products, and services in the real world; this is intended to promote learning in an environment similar to professional practice modern and multidisciplinary teamwork.

The purpose of the CDIO study framework is to create a clear, complete, and coherent teaching–learning methodology following the set of objectives currently considered for undergraduate engineering education [6]. In this way, a pedagogical innovation strategy based on the CDIO framework is available, where the primary objective was to implement an elective subject that was part of the curricular mesh of the biomedical engineering undergraduate program [7].

This subject was planned and developed on the basis of the emergence of digital transformation, one of the most influential technology trends such as the IoT, according to Gartner in its annual statistics on the Hype Cycle for IT. The IoT has been a relevant technology from 2017 to the present [8] with Edge Computing and Data observability, a new phase of the internet in which today there are already more objects and devices than people connected to the network on a global scale. This is how these so-called objects, things, or entities have the computing, storage, and communication capacity to collect, process, and distribute information through the network to which they are connected in a bidirectional manner, allowing them to be analyzed for making better decisions and improving some processes. [9].

**Figure 2:**
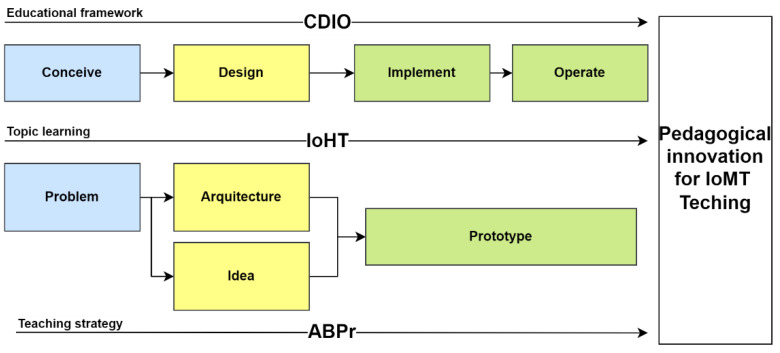
Educational strategies for learning the Internet of Things.

Similarly, in the subject plannig, the integration strategy of problem-based learning (PBL) is proposed, allowing students to simulate real professional contexts, which is achieved due to the design and implementation of structured practices of “real problems in the field of engineering”. Figure 2 presents the framework which intends to respond to significant learning, allowing the development of skills for professional life. These two coupled educational strategies (CDIO-PBL) potentialize the generation of new teaching mechanisms on the application and use of the Internet of Things applied in the health sector (IoHT), a technology currently classified as part of the fourth industrial revolution [10].

This paper presents in detail the development of a methodology for the design and implementation of an elective subject for the biomedical engineering program, taking into account the frameworks and teaching strategies CDIO and PBL for the teaching IoT focused on solving real-world problems in the health sector. The remainder of the paper is organized as follows: Section 2 describes the related works regrading IoT teaching strategies; Section 3 contains the engineering materials and methods to implement our proposed methodology. Section 4 presents the main results of student perceptions and comparisons with related works. Section 5 and Section 6 present the discussion and conclusion obtained from this paper and other considerations for future works.

## 2. Related Works

Some authors have focused on how the fourth industrial revolution has already impacted education in its different curricular aspects. In response to this revolution, Education 4.0 has emerged, gaining strength every day and generating initiatives for teachers interested in this subject [4]. Some works have emerged around Education 4.0 from perspectives such as skills, knowledge, or their involvement in the work of the near future [11,12]. For example, Fisk (2017) analyzed nine trends related to Education 4.0 as follows [13]:

1. Learning happens anytime, anywhere
2. Learning is becoming more personalized
3. The student determines how wants to learn
4. Learning is increasingly project or PBL
5. Learning is more practical through experiences or real cases
6. There is exposure to the analysis and interpretation of data
7. New forms of evaluation emerge
8. Students participate in the design and updating of study plans
9. Students are becoming more independent and teachers more facilitators

The 4.0 revolution implies changes in educational paradigms since it requires the implementation of strategies that promote greater individualization, virtualization, and interdisciplinary strengthening. Likewise, the importance of project-based education and the use of virtual platforms to achieve equal and flexible access for multiple users is highlighted, as presented in different research initiatives, which are presented in Table 1:

Researchers from the University of Phoenix explored the effects of emerging trends that transform society and the global market. They reported the design and innovation process with transformative trends focused on health and technology [11]. Diwan studied the ideal approach for learning topics related to the fourth industrial revolution, such as intelligent technology, the IoT, and robotics, among other technologies. The research focused on preparing future professionals for a world where these cyber–physical systems prevail in all industries, as well as how to develop skills in teamwork and technical communication for new challenges [12]. Fisk analyzed the need for “Industry 4.0”, where humans and machines align to enable new possibilities for technology-based solutions. In addition, the mutual collaboration between the industry and the academy, and time management are topics that the academy encourages to teach in the classroom so that students may use them in their future academic careers [13].

Other researchers reported the initiative to generate new initiatives on the technology of the Internet of Things seen from an ethical and responsible approach is defined since this technology is responsible for the acquisition of data and the flow of information, which suggests that one goes beyond the limits of research and education [14]. Finally, other authors addressed the fundamental steps to building a solid architecture, encompassing the theory, concepts, and implementation of each element integrated into an IoT solution— key aspects to adopt the best practices when guaranteeing reliability and scalability through the communication systems used, security, and data analysis in one’s infrastructure [15].

Although PBL is more suitable for teaching engineering, the need to set up requirements for specific populations and issues is vital before the PBL method moves forward in a class [16]. In addition, Araújo et al. reported the importance of a proper methodology to ensure good outcomes in the classroom and the engineering programs [17]. In contrast, other authors modified the curriculum with topics relevant to the industry and to train people as IoT specialists, but through interviews and industry needed to adjust the teaching process [18]. Some authors innovate with the integration of two or more teaching methodologies. Edström and Kolmos studied the importance of combining PBL and CDIO methodologies as complementary models for teaching basic engineering courses [19]. Tamaki et al. documented the need for integrating PBL with active learning to ensure hands-on training programs and new educational methods to bring academia closer to industry [20]. Finally, Svane et al. reported other methodologies such as Bloom’s taxonomy (especially the revised set), and Knowledge-Skills-Abilities (KSA) combined in a snowball strategy, but the main limitation is related to the time needed to obtain fair results [21]. However, the choice of the best teaching strategy is not an easy task, and it requires interdisciplinary research, the implementation of different new alternatives, and the follow-up of preliminary results.

## 3. Materials and Methods

### 3.1. Implementation of the CDIO Syllabus and the Subject Planning

This section describes how the Internet of Things course was planned and developed, focusing mainly on health applications given the environment where the biomedical engineering program is developed at the Universidad del Rosario (EMCS). Likewise, this document presents an innovative environment to the academic and research community where they can find the main topics covered by the Internet of Things technology for the generation of projects and applications in the health sector.

In this sense, the application of the CDIO educational framework refers to the syllabus or study plan of an engineering program [22]. This educational framework is based on the assumption that newly graduated engineers must be able to solve problems in the real field of the industry area where they work; consequently, they must be educated under the “engineering problem-solving project” paradigm. Indeed, one of the first tangible educational frameworks to achieve this paradigm of engineering education was the CDIO educational framework.

**Figure 3:**
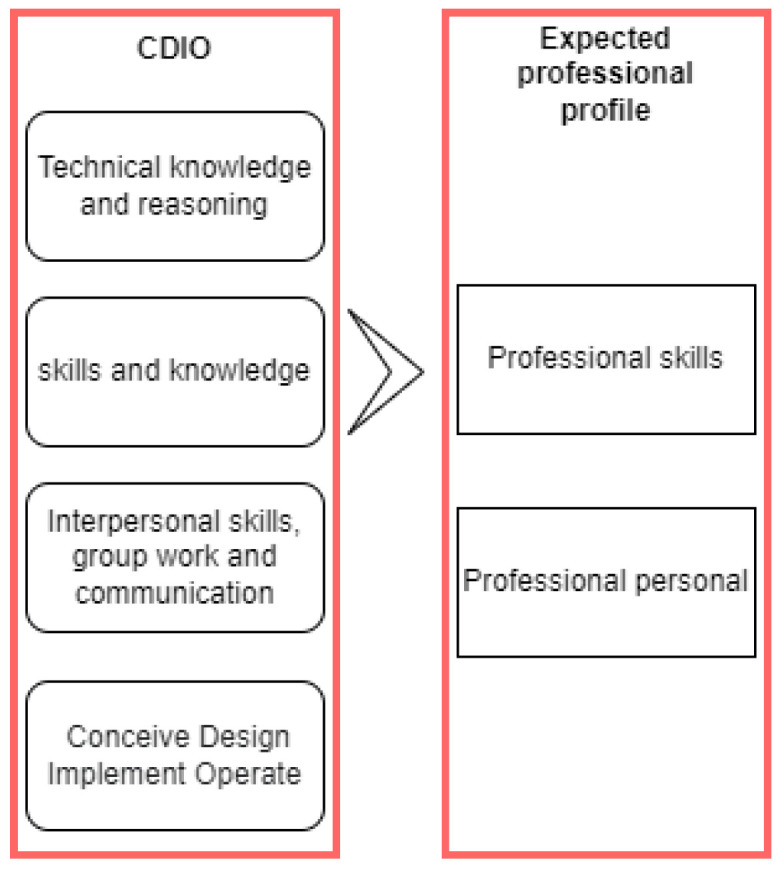
Integration of the expected profile concerning the CDIO syllabus.

Figure 3 shows the expected professional profile once the IoHT elective subject has been seen, which bases its syllabus on the CDIO educational framework, having as its main milestones: technical knowledge, professional knowledge, interpersonal skills, and the educational framework implemented in an actual context; where in each one, these milestones emphasize professional and personal skills.

Although the CDIO guidelines have been successfully implemented in many universities around the world, demonstrating their validity [23], they still need to be validated in the context of biomedical engineering, more specifically in IoT teaching. On the other hand, the motivation to create the subject arises from the need to implement subjects and strategies for teaching and executing new technologies in the engineering curriculum. Therefore, the initiative is taken to design and implement the IoHT subject in the biomedical engineering program.

Regarding the conceptual elements and the approaching of the subject topics through pedagogical strategies of problem-based learning (PBL), applying IoT constitutes a pedagogical novelty, since it is a didactic method that is based particularly on discovery learning and construction where a process of information search, analysis, and organization of ideas is appropriated to solve problems present in the health sector [24]. On the other hand, the role of teachers is established as a counselor, where they challenge the student to seek practical solutions and, with a high component of ingenuity, guide them to problems or situations in the field of clinical engineering, always pursuing the objective of learning to learn and to solve problems. The implementation of projects based on IoHT will set a step forward for the biomedical engineering program students for the revolution of things in the different fields of technology of the so-called 4.0 revolution, based on realistic trends in science and engineering [25].

### 3.2. Description of the Subject Content

The subject development contains 14 modules, and each one of them was developed under a structure of eight sections. In the first section, the expected learning results (RAE) are presented. In the second section, students are presented with a brief description of the materials needed to develop each of the exercises. In the third section, the general concepts of the topic to be addressed are masterfully exposed, where the relevant information on the use of software and hardware is contemplated per the content of the module. In the fourth section, a problem in the area of health is contextualized, helping students to understand and assimilate the importance of generating solutions for the exercise of their profession, under situations similar to those that they will have to face during professional practice.

**Figure 4:**
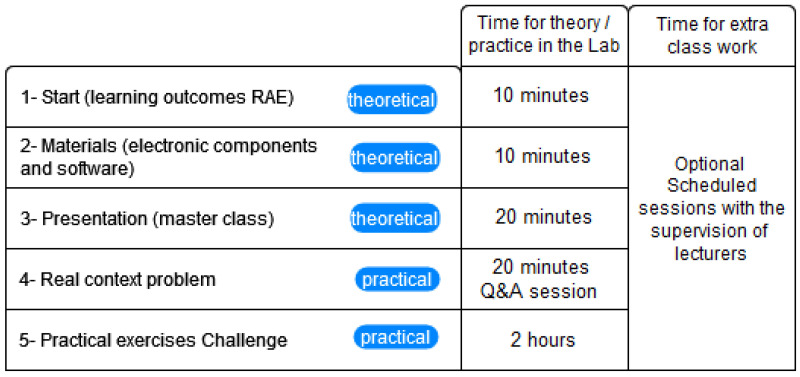
Pipeline reporting the modules’ structure proposed.

In the fifth section, different practical exercises are presented where the student is expected to acquire knowledge and skills from a basic to a complex level that demands rehearsing and testing programming knowledge to the point of documenting and exploring new alternatives combining software and hardware technology. Finally, a challenge is left based on the presented content of the module, in such a way that the student is induced to search, study, and apply the theme of the information of the module presented. Figure 4 depicts each of the sections of the 14 modules of our subject with a dedicated time of 3 h for the whole module. In addition, Figure 4 presents the activities and the time, but with an option to schedule tutoring hours if they need additional supervision or the lab setting to test or finish the assignments of the lab guides proposed.

**Figure 5:**
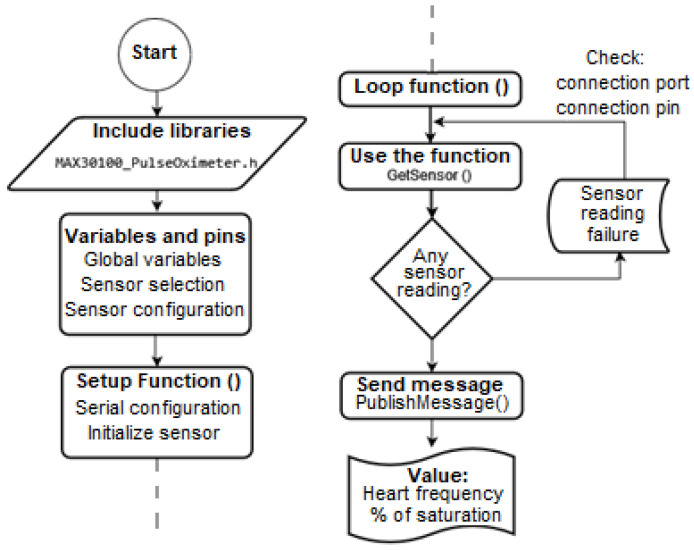
Pipeline for the assignment related to IoMT applications.

The laboratory guides cover the development of programming and hardware assembly activities that are particularly challenging for students. In particular, the designed algorithm for one of the guides with greater student interest is presented in Figure 5. Specifically, the guide deals with IoMT applications, where biomedical sensors are used to capture cardiac signals, pulse oximetry, electromyography, and electrodermal activity. In that order of ideas, the flowchart of one of the exercises proposed in the IoMT application guide is presented, where the pulse oximeter and heart-rate sensors for Arduino were used for the acquisition of physiological variables through microcontrollers. In this way, the main goal of the sessions is centered on the use of electronic devices (sensors/microcontrollers) widely used in real-world situations. Moreover, our students may be motivated to develop initiatives in the area of biomedical engineering with the integration of the Internet of Things applied to the medical field.

**Figure 6:**
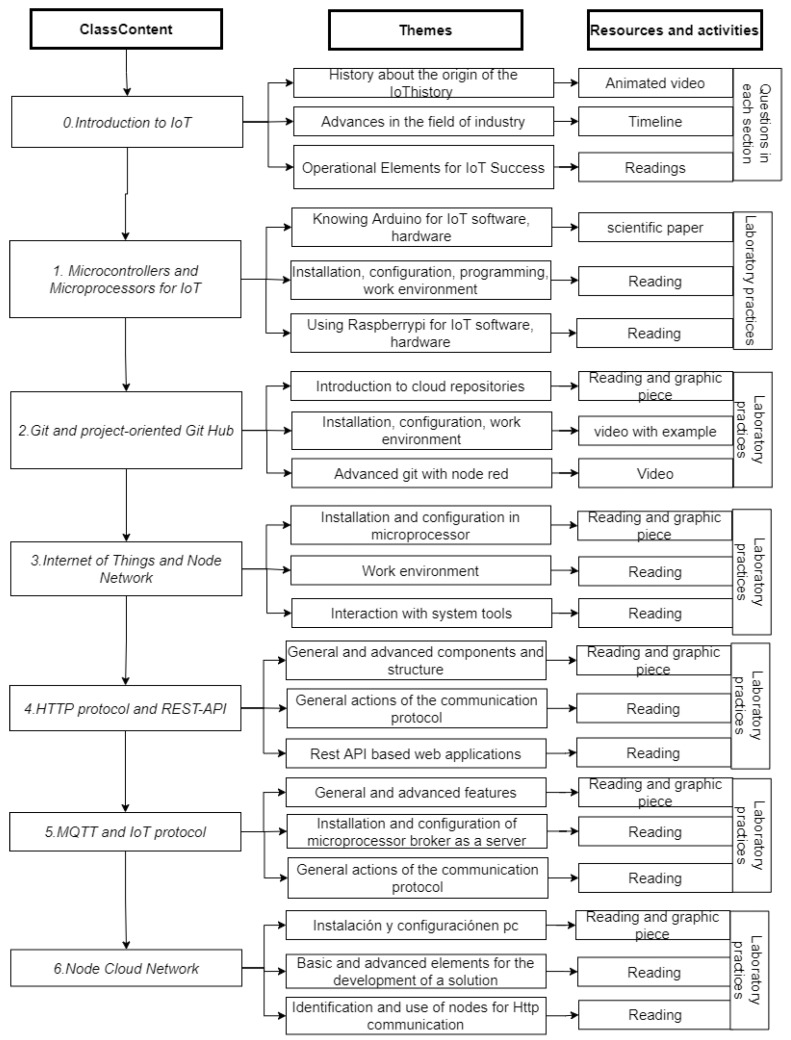
The content structure of the subject from Modules Zero to Six.

**Figure 7:**
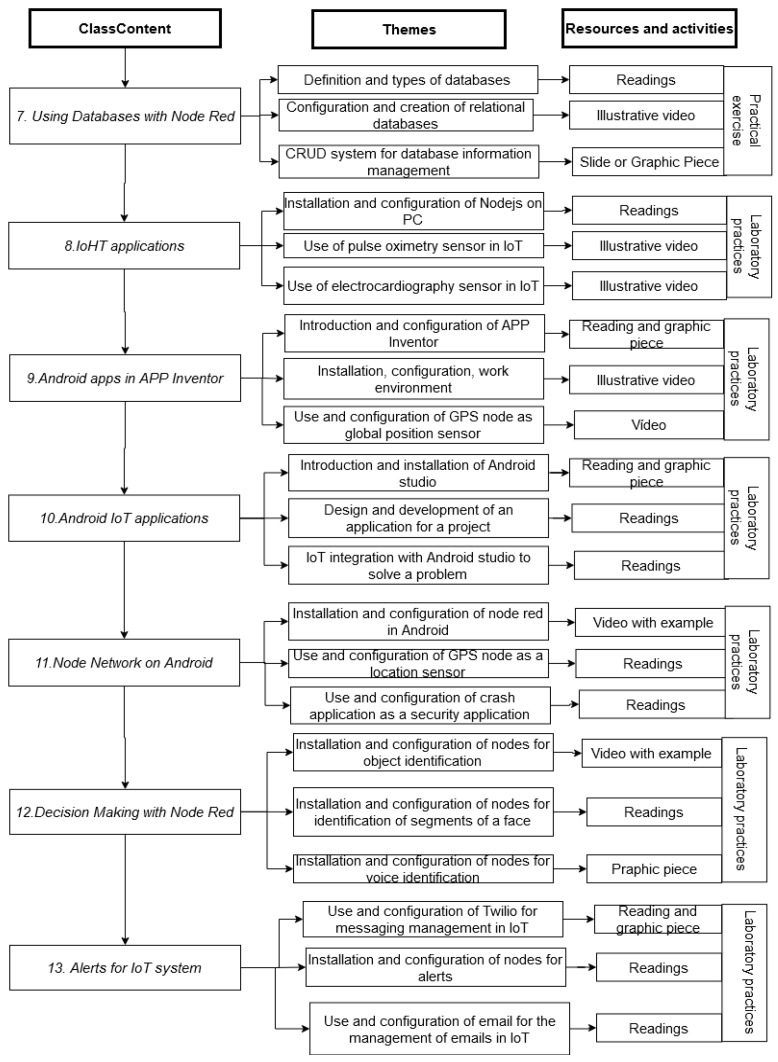
The content structure of the subject from Modules Seven to Thirteen.

One of the most important components of the design and development of the subject was to be able to address knowledge from the “zero” module, allowing an introduction to IoT technology. Module One deals with basic concepts that allow students to become familiar with the different open-source hardware and software platforms, where they will learn how to correctly install and configure the most-used development programs and cards in IoT, as well as basic and advanced programming command structures; Figure 6 and Figure 7 show in detail the description of the modules.

The Git and Git-Hub module is guided toward enabling students to manage software projects. This module covers collaborative work tools for the management of programs and projects related to software development.

Module Three allows students to use software for the design of Internet of Things systems such as Node-Red [26]. This module allows students to have advanced knowledge of the Node-Red platforms together with the main programming cards, allowing them to practice the different types of wired communication, such as UART, I2C, and SPI.

Module Four covers the HTTP and HTTPS protocols. This module details the main characteristics of the HTTP protocol and applications under the use of API, which allows students to become familiar with the different concepts and structures of programming commands for information transfers between objects using wireless communication protocols.

Module Five refers to the MQTT Protocol. In this module, the MQTT protocol is applied, which allows students to become familiar with the wireless information transfer protocols between M2M machines [27].

Module Six presents the management of the Node-Red platform in the cloud. This module allows knowing and interacting with the development platform where IoT systems can be generated, as well as their management with Node-Red, together with the different connected devices.

Module Seven explains relational and non-relational database management. This module allows students to learn about a management and query system from a database, based on structured query languages (SQL) and unstructured query languages, such as JavaScript. Storage in IoT is important since information can be managed on a web development platform; it is also the basis for the implementation of decision methods in the cloud using technologies such as big data or machine learning.

Module Eight contains practice on the use of biomedical sensors; in this module the student can send different physiological variables such as electrocardiogram (ECG), electromyography (EMG), skin conductance, and oxygen saturation (SpO2) through the implementation of a monitoring system under the technology of the internet of things in health IoHT.

Module Nine integrates Android Application development in APP Inventor. In this laboratory, different applications are focused on the health area (pulse oximetry, electromyography, electrocardiography, and electrodermal) from a Smartphone where the attributes with which these devices are developed can be used.

Module Ten integrates and runs Android IoT Applications. This module is integrated with different applications focused on the health area (pulse oximetry, electromyography, electrocardiography, electrodermal), from a Smart Phone where physiological signals can be displayed in real-time, in addition to performing the respective storage and interpretation of each of the signals.

Module Eleven covers Android Applications with Node-Red. This module works with Node-Red in conjunction with the Android Studio operating system, making use of the different sensors with which these devices are developed.

Module Twelve was designed on the basis of the use of interactive analysis tools. In this module, the data acquired and stored in the server is used, from the development of the different applications where the sensors were used as a source of information acquisition that allows decision-making from a particular event.

Module Thirteen deals with alert handling for IoT Systems. This module deals with different strategies on how to generate alert systems and interoperability with everyday tools, such as text messages and email. It provides the opportunity to learn the practical use of Node-Red for the generation of alerts in IoT systems [28].

Finally, as a closing of the subject, a final project is promoted with navigation algorithms in robots simulating a realistic environment. In this maker space, the student may use Makeblock educational robots and their different sensors to develop ideas based on IoT [29]. Then, students work together under the supervision of the teachers in the implementation of an original application oriented to the solution of a particular problem in the health sector.

### 3.3. Materials Used for the Development of Each of the Modules

**Figure 8:**
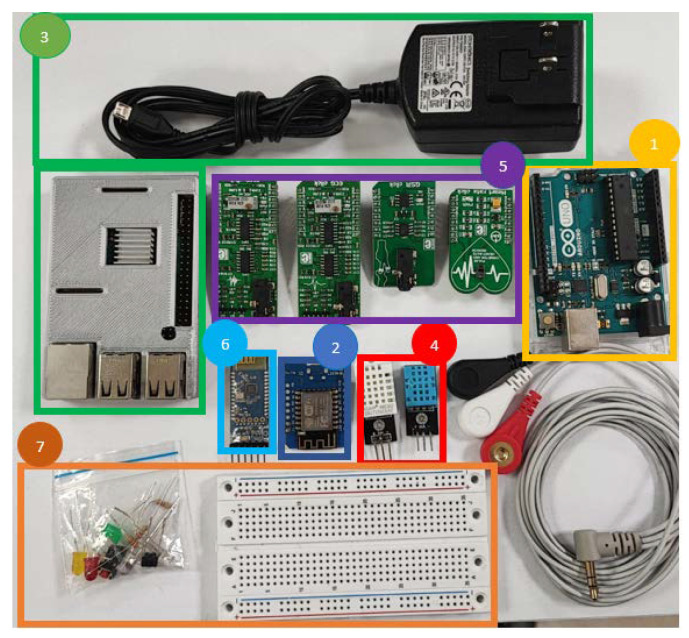
The materials and devices used to implement the all modules.

For the development of each of the modules proposed in the subject, a work kit was defined, which contains the different components and electronic cards necessary to carry out each of the exercises. The educational kit is made up of the following materials, and it can be seen in Figure 8:

Some important characteristics to take into account in the use of sensors are listed below:

- Item 1: Arduino UNO board (connection cable)
- Item 2: Wemos D1 R1 mini card (connection cable)
- Item 3: Raspberry pi 3 microprocessor (cables and adapter)
- Item 4: Temperature and humidity sensors (DHT 11/22)
- Item 5: EMG, ECG, and heart rate sensors (Click Board/MIKROE)
- Item 6: Bluetooth module (HC-05)
- Item 7: Protoboard, some jumpers and some Light-Emitting Diodes (LEDs)

Temperature and humidity sensors (DHT 11/22) cover ranges from −40 to +125 degrees with a relative error of 2 to 5%; in the case of relative humidity, ranging from 20 to 80% with a relative error of 5%.

For biomedical EMG, ECG, and pulse oximetry sensors (click Board/MIKROE), according to their specification in the data sheet: The click EMG measures electrical activity produced by skeletal muscles. It contains an MCP609 operational amplifier and MAX6106 micro-power voltage reference. EMG click is designed to work with a 5 V power supply. The Click Board/MIKROE has an analog output (AN pin).

The MCP609 Features Microchip Technology Inc.’s MCP606/7/8/9 belongs to the family of operational amplifiers (OP AMPS) with stable unity gain and low offset voltage (250 µV, maximum). Performance characteristics include rail-to-rail output swing capability and low input bias current (80 pA @ +85 °C, maximum). On the other hand, the MAX6106 features a low-cost, low-dropout micro-power voltage reference (LDO). This three-terminal reference is available with output voltage options of 1.25 V, 1.8 V, 2.048 V, 2.5 V, 3 V, 4.096 V, 4.5 V, and 5 V. For this click, we use 2.048 V.

The ECG contains a 3.5 mm jack to which three electrodes are connected. The electrodes will detect the electrical activity of the heart and send the signal to the 3.5 mm jack. After that, the signal needs to be amplified and filtered, so the click also contains two amplifiers, two high-pass filters, and one low-pass filter. Once the data is acquired, amplified, and filtered, it is sent to an analog pin. Then, the microcontroller reads this analog data and converts it to digital, this digital data measures the value of the electrical activity of the heart at that particular moment.

The heart rate sensor is suitable for prototyping wearable devices for fitness or medical applications. The MAX30100 is a low-power device IC and also incorporates ambient light, cancellation, and motion artifact resiliency. For best results, readings should be taken through the fingertip (the combination of red and IR LEDs is optimized for this application). Readings, however, can be adversely affected by excessive movement and temperature variations. In addition, too much pressure can constrict capillary blood flow and decrease the reliability of the data.

The advantage of delivering this educational kit for teaching IoT as a loan is to use it anywhere and anytime, either in the bio-instrumentation laboratories of the Universidad del Rosario of the biomedical engineering program or remotely in an environment of collaborative work for the development of each of the proposed modules. During the pandemic, the delivery of these kits to students by courier was of help for the development of classes.

From a more general perspective, each of the modules begins with a problem framed in a real context within the framework of biomedical engineering, in such a way that the work methodology begins with the identification of key factors that allow a solution to be addressed efficiently and considering all the information necessary to generate a comprehensive solution. That is why a template for the design of the architecture of a solution based on IoT technology was designed and implemented (https://iot.urosario.edu.co/dise%c3%b1oiot/ (accessed on 15 November 2022). This template includes the following items:

**Solution description:**

Name of the solution/problem to solve/scope/results

**Requirements:**

Necessary materials

**Entities:**

Variables (name/type/range)

**Services:**

Functions (description/arguments)

**Events:**

Functions (description/arguments/response)

**Architecture:**

Devices to connect (sensors/actuators/devices)

**Connection protocols:**

Considerations (selected protocol/features)

**Architecture:**

Architecture type (centralized/distributed)

**Layers description:**

Number of layers (name/description of each one)

**Storage solution**

Storage type (relational/non-relational/considerations)

The main goal is that students acquire a complete conception and analysis of problems through a flexible design framework adapted to any kind of need or development of IoT systems. Then, we hypothesized that students would carry out the analysis process before facing the development of algorithms and programming of electronic cards.

## 4. Results

The development of the contents of the subject has focused on each of the aforementioned statements, aligning the contents of the IoT technology in each phase of development. Moreover, it was based on the competencies declared in the CDIO syllabus, such as technical knowledge, skills, personal and professional attributes, interpersonal skills (communication and teamwork), and the ability to conceive, design, implement, and operate systems in the context of organizational and social environments.

The contents of the subject were designed, developed, and implemented on a web page, https://iot.urosario.edu.co/cursoiot, addressing the themes of each phase of development for an application in IoT; integrating each of the statements of the CDIO syllabus. In this sense, what was sought was for the student to acquire the skills and knowledge that the future engineer needs in real scenarios, based on the development of each of the modules of the subject, which were previously reviewed and executed to be completely sure that each of the exercises works correctly.

**Figure 9:**
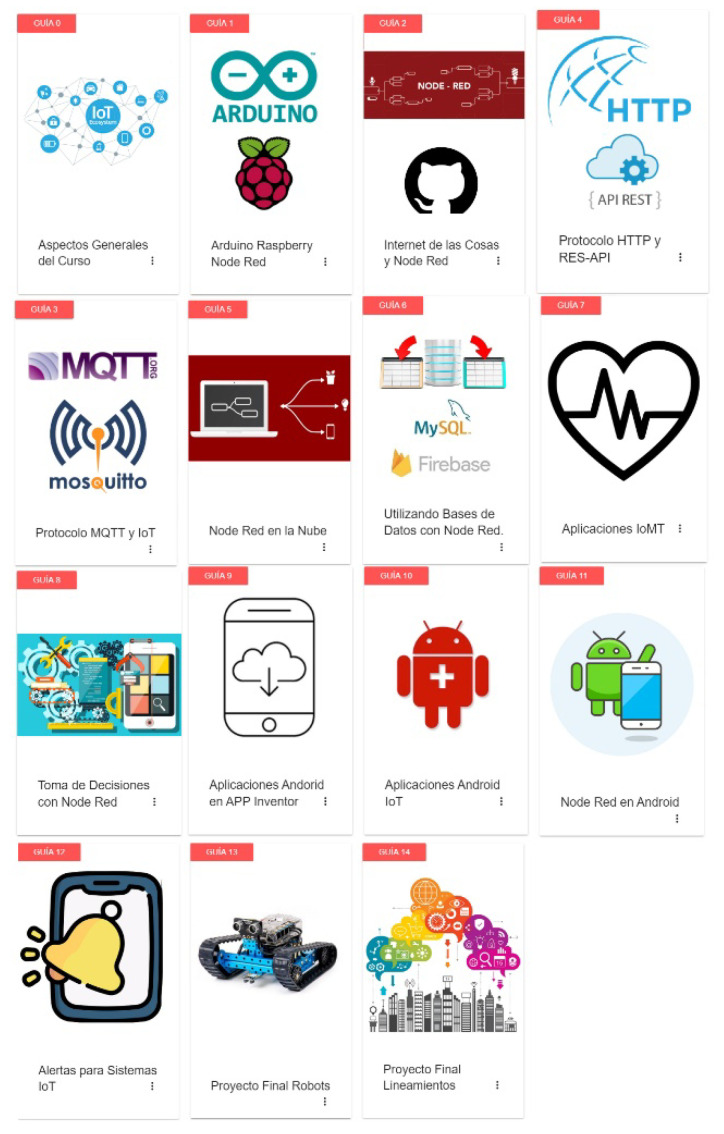
The users’ visualization of the subject Modules from Zero to Six.

Within this framework, as can be seen in Figure 9, the design of the modules is presented in a clear and detailed way, guiding the students step by step in the development of each of the proposed activities.

The course developed fourteen modules that contain different topics for teaching IoT. In this way, theoretical and practical points are raised in each one that focuses on the solution of a particular problem in the health sector through the use of IoHT.

### Planning Learning Outcomes

According to the characteristics of the subject, Table 2 presents the design and implementation of the methodology planned for the development of each of the modules in terms of credits, number of practical and theoretical hours, and the expected learning objectives at the end of each module.

**Figure 10:**
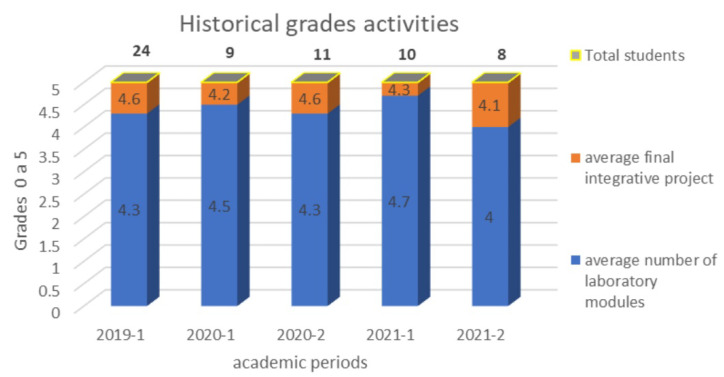
Average grades of undergraduate students in the last five academic periods.

To date, our methodology has been taught at the undergraduate and graduate levels with approximately 62 undergraduate students and 10 graduate students, with high average scores in the upper 80% (>4.0/5.0) and with the desertion of only 2 students, as presented in Figure 10. As a consequence, multiple end-of-course projects have been generated with applications to the medical and clinical industry, allowing the use of IoT technology to be encouraged as a component of innovation in the different services. Similarly, some students have taken the knowledge about this technology and formalized enterprises as micro-entrepreneurs in Colombia, generating job opportunities in the healthcare area as a life project. In this way, engineering and technology can be seen as a bridge to optimize the production capacities and improvement of current systems in the different fields of production and services.

The students’ perception concerning the experience of the IoT subject was focused mainly on the usefulness of the knowledge acquired and the multiple applications it has in the field of biomedical engineering, with the main objective being the social impact and the improvement of manual processes. On the other hand, a key and better-valued aspect was the attention and support of the teachers at all times, as well as the teaching methodology. Likewise, they highly valued the practical component with which the subject is designed, where 70% are practical in the laboratory and 30% are theoretical. However, they suggested some improvements about the use and configuration of some programming cards and more space in the laboratory to practice the different exercises proposed and the accompaniment or advice in the generation of the course final projects.

## 5. Discussion

The adoption of new technological trends such as industrial revolution 4.0 requires knowing some issues regarding sustainability and innovation in engineering. Hence, in the steady teaching process, it is essential to propose new methodologies integrated with the novelty technological developments. These new methodologies may lead to relevant transformation and social impacts in the different fields of what the biomedical engineer performs.

Regarding the hyper-cycle in 2021 by Gartner, IoT presents high importance for new developments and applications in different industry fields. In this sense, we found an outstanding opportunity to train students from universities and research centers to face several challenges that the future holds in the short term. Consequently, the university tends to be relevant in the continual industrialization and generation of knowledge of issues, and proposed solutions are currently provided.

The industry plays a vital role in how the focus changes in subject guides. These changes are associated mainly with the need that students have to strengthen their profile before they are faced with a real problem. Therefore, the requirements of a specific issue are often challenging, especially when there is a software component, device development, and generation of sustainable architectures.

Similarly, the traditional models of education and the new teaching initiatives in engineering require constant adaptation and reforms concerning the current needs of the labor market. This research answers the purpose of improving critical thinking skills oriented toward the application and solution of real problems. Indeed, students have to identify strengths and weaknesses in affinity with the pedagogical strategy used in the teaching and learning process of the IoT subject.

According to the main results reported by Almada-Lobo [13], the context of CDIO applied to engineering must be flexible enough to integrate different concepts and roles when addressing a problem. Moreover, it also suggests the consolidation of fundamental principles under test environments to ensure a proper solution. The design of a prototype that solves a specific problem requires the integration of conceptual and practical prerequisites, which are motivated by the teaching process.

Although our proposed methodology was tested in the face-to-face modality for the first time, due to the pandemic, the proposed teaching strategy was able to be adapted for virtual and hybrid modalities with significant acceptance and perception by students, as presented in Figure 10. In the present semester, 23 students were enrolled and participated actively in our teaching activities. The last five years are summarized with the following results: the employment relationship of students in large and remarkable hospitals in Colombia; the conception of two small and medium enterprises in the healthcare sector; and finally, the first steps to the creation of an IoHT technology-based spin-off pioneer in Bogotá and Colombia.

In addition, there are still some key aspects to improve in future works. For example, the implementation of IoT-proposed solutions in areas of biomedical engineering where engineers may perform in a real-work context. The deployment of IoT technology in different industry environments is increasing exponentially, with web or mobile applications showing the fastest growth in each of these environments. Thus, the 4.0 revolution implies changes in paradigms of traditional education, since it requires the generation of strategies that promote greater individualization, virtualization, and interdisciplinary strengthening.

Likewise, the importance of problem-based education and the use of virtual platforms to achieve equal and flexible access for multiple users are emphasized by other researchers [30,31]. Our proposed educational modules consider both components of social and face-to-face learning and virtual learning. The latter seeks the development of collaborative skills that, aligned with creative and supportive thinking, facilitate independent work strategies with a high level of intrinsic motivation, as well as the availability of the teacher as a learning facilitator and extrinsic motivator.

## 6. Conclusions

The creation of the Internet of Things, IoT, subject provides the student with a general and very well-structured overview of the practice of this IoT technology in biomedical engineering. Moreover, it is possible to implement the method of work from a problem of a real environment, the development of an application from the requirements, entities, architecture, and the own interface of each solution in direct agreement with the CDIO initiative and PBL.

The implementation of the CDIO initiative through the IoT subject has been highly rated by the students, since 70% of the classes are developed under laboratory practices, allowing their knowledge to be compared among the members of the workgroup. This experience undoubtedly leaves a complete and focused learning that fosters new issues, which opens new challenges associating knowledge and academic and personal skills with one’s future professional and work development interests.

## Figures and Tables

**Table 1:** Summary of related works on IoT Education Initiatives.

Goals	Methodology	Pros
Transdisciplinary, intercultural competencies and social intelligence skills [11]	Use of gaming platforms for group collaboration	Prospective methodologies from the Delphi technique
Traditional education with readings and learning environments [12]	Use of web resources and inter-institutional groups	Active collaboration and learning methodologies with virtual interaction
Personalized and repetitive frameworks for education [13]	Permanent learning and peer learning with teachers more as facilitators	Interaction between human and machine, using technology and its benefits
Raise awareness among young people regarding behaviors and habits in the use of energy and natural resources [14]	Teacher’s guide under a real environment in terms of scalability, responsiveness, and simplicity through elements based on the web, mobile, forums, and others	Use of open-source technologies and services with app-based solutions used for educational purposes
Previous knowledge/experience with technology applications [15]	Practical workshop and experimental research in a mobile application development course	Use of App Inventor and interdisciplinary work

**Table 2:** Learning outcomes description for the course with credits, lesson planning, and RAEs.

Credits	Lesson Planning	Expected Learning Outcomes (RAEs)
3	Class 20% Lab practice 80%	Implement mathematical models through axioms, theorems, and laws, as well as analyze the results from the field of mathematics. Solve problems, design processes, and carry out experiments typical of biomedical engineering using computer tools for the capture, processing, simulation, and visualization of information. Develop algorithms that are coded, debugged, and executed in graphical programming environments using structured programming methodologies and stream-based programming that solve specific tasks typical of the application of IoHT systems.

## Data Availability

Not applicable.
